# Dual-Target Radiosurgery for Concomitant Continuous Pain Presentation of Trigeminal Neuralgia: Radiomodulation Effect and Dose

**DOI:** 10.7759/cureus.51602

**Published:** 2024-01-03

**Authors:** Alejandra Moreira, David A Santos Hernández, Victor Caceros, Kaory C Barahona, Fidel Campos, William A Reyes, Alejandro Blanco, Tatiana Soto, Juliana Ramirez, Ricardo Mejias, Claudia Cruz, Eduardo E Lovo

**Affiliations:** 1 Neurosurgery, International Cancer Center, San Salvador, SLV; 2 Radiosurgery, International Cancer Center, San Salvador, SLV; 3 Radiation Oncology, International Cancer Center, San Salvador, SLV; 4 Radiation Oncology, International Cancer Center, Diagnostic Hospital, San Salvador, SLV; 5 Radiosurgery, International Cancer Center, Diagnostic Hospital, San Salvador, SLV; 6 Radiosurgery, Robotic Radiosurgery Center, San Jose, CRI; 7 Radiation, Robotic Radiosurgery Center, San Jose, CRI; 8 Radiosurgery, Centro de Radiocirugia Robotica, San Jose, CRI; 9 Medical Physics, Robotic Radiosurgery Center, San Jose, CRI; 10 Anesthesia and Pain Management, Hospital De Diagnóstico, San Salvador, SLV

**Keywords:** modulation, radiosurgery, atypical pain, concomitant continuous pain, trigeminal neuralgia

## Abstract

Objectives: Patients with trigeminal neuralgia (TN) experience concomitant continuous pain (CCP) that can be difficult to treat. A dual-target approach delivering a high dose of radiation to the nerve and the contralateral thalamus can develop a fast radiomodulation effect on lowering pain. We sought to determine if this effect was dose dependent.

Methods: We retrospectively reviewed 21 patients treated with radiosurgery in CCP and severe TN pain, with a visual analog scale (VAS) score of nine out of 10 at the time of treatment. Ten patients were treated with a high dose (>120 Gy) in the thalamus 90 Gy to the nerve, and the rest with a low dose (<120 Gy) to the thalamus and >90 Gy to the nerve.

Results: Of those who received the high dose to the thalamus, six patients (60%) received 140 Gy, and four (40%) received 120 Gy, with a median dose to the trigeminal nerve of 90 and 85 Gy, respectively. The high thalamus dose showed a radiomodulation effect from day 1. The low thalamus dose did not produce radiomodulation on any of the first four days. The percentage of VAS score reduction one month after treatment was higher in the high-thalamus dose group than in the low-thalamus dose group. At three months, VAS score was 2 in the high-dose group and 4 in the low-dose group.

Conclusions: The radiomodulation effect in pain and dual-target radiosurgery is dose dependent in CCP in TN; a high dose can provide a more consistent clinical result than a lower dose.

## Introduction

A subgroup of patients with trigeminal neuralgia (TN) experiences concomitant continuous pain (CCP). This form of pain is continuous, dull, burning, or aching in the areas corresponding to the trigeminal nerve [1,2]. The persistent pain aligns with the same distribution as paroxysmal pain, and its intensity can either match or exceed that of paroxysmal pain [3].

Many of these patients have endured TN for years, undergoing various medical treatment regimens or invasive procedures such as microvascular decompression or percutaneous rhizotomy. These patients face significant compromises to their quality of life (QoL), including difficulties with eating and sleeping. Managing their pain presents a challenge, particularly for those who have not found success with invasive procedures.

Radiosurgery (RS), a noninvasive treatment for TN, is a well-known and cost-effective procedure [4-6]. Traditionally, RS treatment for TN involves delivering a high dose of radiation to the trigeminal nerve; however, pain relief is typically not immediate, with time intervals ranging from one to six months [7]. RS is an impractical treatment option during a pain crisis associated with CCP.

Neuromodulation via focal radiation is a well-known phenomenon but poorly understood. For some authors, it is associated with sub-ablative doses, although in the most purest of senses, it would be a clinical manifestation of an alteration of a neuronal circuitry with what is known to be a sub-ablative dose or at a time much before the onset of necrosis occurs as explained by radiobiology.

We proposed a new approach for treating refractory CCP by administering a high dose of radiation, typically 90 Gy, to the affected nerve and 140 Gy to the opposite side of the thalamus in a region that encompasses the centromedian (CM) and parafascicular complex (PFc) during the same treatment session. We achieved neuromodulation via the focal radiation effect in 100% of the patients in this small series [8]. We suggest that radioneuromodulation may provide rapid (i.e., under seven days) and substantial (i.e., 50% or more) pain relief after administering a focal radiation dose to various neuronal circuits in both non-oncological and oncological indications [9].

As radiation time increases, the biological effectiveness of a specific radiation dose to tissue diminishes [10-13]. Achieving the steepest dose gradient in functional RS requires a considerable amount of time regardless of the platform. Linear accelerators and cobalt-dependent machines like the Gamma Knife deliver physical doses at different treatment times, with the latter experiencing more variation due to cobalt decay. Researchers can use the biologically effective dose (BED) to evaluate variables like treatment time and dose, potentially standardizing functional treatments across platforms over time.

Our initial work showed lower doses effectively provided rapid pain relief in oncologic [9] and non-oncologic pain [8]. We aimed to reduce doses to the medial structures of the thalamus for optimal radioneuromodulation, using a lower dose to alter neuronal circuitry hopefully without destroying it [14,15]. We present clinical results comparing a high-dose to a low-dose approach in patients with CCP TN pain crisis. This study is the first to assess different dose strategies in dual-target irradiation for a specific clinical manifestation of TN.

## Materials and methods

This retrospective cohort study, conducted from September 2019 to September 2022, involved 21 patients with TN CCP pain that persisted for at least four weeks and was classified as severe, ranging from eight to 10 out of 10 on the visual analog scale (VAS). These patients experienced a pain crisis and underwent RS using a dual target strategy. A pain specialist doctor or neurosurgeon with experience treating TN deemed all patients' refractory to medical treatment. Treatment modalities had to include first- and second-line treatments, encompassing opioids. Some pain levels were unresponsive to invasive treatment modalities, and no alternatives were feasible or desired. The primary endpoint aimed to achieve radioneuromodulation within five days after treatment by reducing the VAS score by at least 50%.

Before the procedure, we interviewed patients regarding their pain history, including the duration of pain since diagnosis, duration of pain crises, and pain intensity using a numerical rating (i.e., VAS), the Barrow Institute Neurological Pain Intensity Scale (BNI) prior to treatment, and history of previous treatments (procedures and medication). We also administered a QoL questionnaire using the EuroQol 5-Dimensions 5-Levels (EQ-5D-5L). Before discharge, we advised patients to continue taking their regular medications as prescribed. The institution's ethical committee approved the study, and all patients provided written consent. The senior author (a neurosurgeon) conducted or supervised the thalamus and nerve targeting; a radiation oncologist and physicists approved the final plan.

Radiosurgical technique

For patients treated with the GammaRay Infini ((GK); Masep Medical Company, Shenzhen, China), we placed them in a stereotactic frame under local anesthesia and mild sedation. We conducted magnetic resonance imaging (MRI) using a 1.5-Tesla Avanto (Siemens Corporation, Erlangen, Germany) with constructive interference in T1 and T2 steady state and 1-mm slices with no gaps of the head. We then transferred the images to the treatment planning station and treated patients immediately after approving the plan.

For the Cyberknife (CK) platform (Accuray, Sunnyvale, CA, USA), the simulation process involved placing a thermoplastic mask and acquiring a 1-mm computer tomography (CT) of the head. Next, we used a 1.5 MRI General Electric (General Electric, MA, USA) device for image acquisition, obtaining a 1-mm T1 MRI with and without contrast and fast imaging employing steady-state acquisition (FIESTA). We typically treated patients 48 to 72 hours after planning.

We identified the anterior and posterior commissures (PC) in the axial plane, we drew the intercommissural line (ICL), and measured the distance from the PC anteriorly along the ICL, typically 4 mm, labeling it as Y. Then, we drew a 90-degree angle from the PC to Y along the ICL and determined Z, typically 4 mm above the ICL. We set the X coordinates four to 5 mm lateral from the contralateral thalamic border to the side of the pain. Using a 4-mm collimator with GK and five mm for CK, we placed a single shot and administered 80 to 140 Gy as a maximum dose (Dmax), depending on the low-dose or high-dose strategy. Finally, we prescribed an 80 to 90 Gy Dmax using a single 4 mm (GK) or 5 mm (CK) isocenter positioned in the retrogasserian zone of the affected nerve.

We based pain evaluation on the Initiative on Methods, Measurements, and Pain Assessment in Clinical Trials (IMMPACT) recommendations [16] and assessed patients using an 11-point VAS with zero representing “no pain” and 10 meaning “pain as bad as you can imagine.” We classified cases as having a radioneuromodulation effect if they experienced a nontransient pain reduction of at least 50% within the first five days after treatment. We evaluated QoL using the EQ-5D-5L, as previously mentioned. We followed up with patients 24, 48, 72, and 96 hours after treatment, at 15 days and one month after stereotactic RS (SRS), and every month thereafter or sooner if the patient reported pain.

Regarding dose and radiomodulation, we grouped patients according to the dose administered to the thalamus and performed statistical inferential analyses using the following differentiation. We used the subgroups of clinical response and radiation doses to the nerve and thalamus for descriptive statistical analysis. To catalog patients' responses, we further classified them as 1) early radioneuromodulation: a sustained reduction ≥50% of the initial VAS score 24 hours after treatment; 2) late radioneuromodulation: a reduction ≥50% of the initial VAS score between 24 or 96 hours after treatment; 3) pseudoradioneuromodulation: an initial reduction (≥ 50%) of their VAS score that was not maintained during the first five days after treatment; and 4) no radioneuromodulation: not achieving a reduction ≥50% of the initial VAS score five days after treatment. We classified the different dose strategies as high dose (>120 Gy to the thalamus) and low dose (<120 Gy). For the nerve, we considered 90 Gy a high dose and <90 Gy a low dose.

Calculation of BED

For GK SRS single continuous exposures, we calculated the BED using the equation published in the following reference [11], where DT represents the total physical prescription dose at the 100% iso-dose, given as a single continuous exposure. The a/b ratio is a tissue-specific constant with a value of 2.47 Gy in this study. The term j(X,m) is a function of the protocol and the repair rates; this function mediates the effects of the dose rate and the exposure time, “m1” and “m2” are parameters that represent two sublethal radiation damage repair rates associated with prolonged exposure, and “c” is the partition coefficient of the slower component (m1 > m2). The absolute partition between the two repair processes “m1” and “m2” is 1.0/(1+c) and c/(1+c), respectively. To avoid confusion with the physical dose, we use the units Gy 2.47 for BED values. These units account for the modulation by the α/β ratio used in the calculations.

Statistical analysis

We performed all analyses using Jamovi 2.2.5, ensuring compliance with normality and homoscedasticity, assumptions. We evaluated quantitative variables with the Shapiro-Wilk and Levene’s tests. For variables that met these assumptions, we presented them with means and standard deviations, while we reported other variables with medians and interquartile ranges.

We compared high-dose thalamus and low-dose thalamus patients using unpaired t-tests or Mann-Whitney U tests for quantitative variables, and Chi-squared or Fisher’s exact tests based on expected results in contingency tables. To correlate continuous variables, we calculated the percentage reduction in VAS score during the first five days after SRS ((VAS score in the period/initial VAS score) - 1) and obtained the thalamus and nerve dose using the Pearson coefficient.

We assessed differences between the groups in percentage reduction of VAS score and QoL index during the first five days after treatment, using repeated measures analysis of variance (ANOVA) with Fisher’s least significant difference method for post-hoc analysis. We evaluated differences at one and three months using unpaired t-tests for quantitative data and Fisher’s exact tests for categorical variables.

We analyzed adverse events and the need for additional procedures using Fisher’s exact tests, ensuring all assumptions were met. Due to the sample size, we conducted analyses for subgroups concerning nerve-thalamus doses with descriptive statistics.

## Results

**Figure 1:**
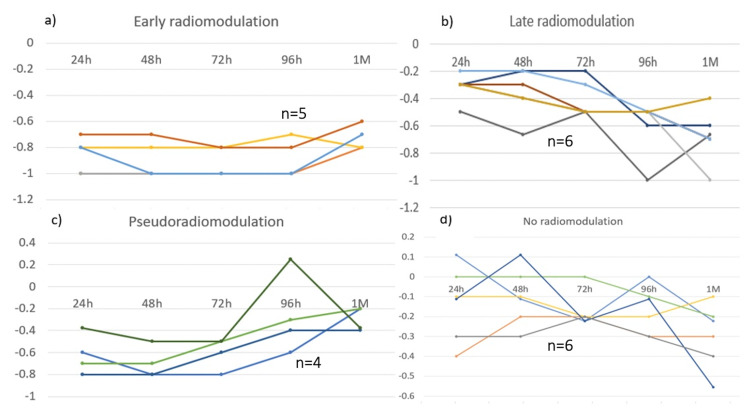
Patterns of reduction of pain after SRS in patients with trigeminal neuralgia Abbreviation: SRS, stereotactic radiosurgery

Table 1 summarizes the patient and treatment characteristics while Figures 1A-1D present the patterns of radioneuromodulation response. Of the 21 patients studied, 16 (76.2%) received GK treatment, and five (23.8%) received CK treatment. Ten patients (47.6%) received high-dose radiation therapy to the thalamus, with six (60%) receiving 140 Gy and four (40%) receiving 120 Gy. The median dose of radiation therapy to the trigeminal nerve was 90 Gy for those receiving 140 Gy to the thalamus and 85 Gy for those receiving 120 Gy to the thalamus. Among those treated with a low dose to the thalamus, four received 110 Gy, four received 90 Gy, and three received 80 Gy. Most of the patients were women (n=15; 71.4%), and six were men (28.5%). Patients had a relatively long history of TN (84 months, range, 6 to 396); 16 patients (76.2%) had a previous history of percutaneous rhizotomy or surgical treatment. The mean duration of CCP prior to treatment was 65 days (range, 30 to 150). All patients were in a pain crisis, with a VAS score of nine out of 10 (range 8, 8 to 10), and BNI scale of IV and V.

**Table 1:** Patient and treatment characteristics compared according to the dose delivered to the thalamus All patients were in crisis at the time of treatment. P<0.05 indicates statistical difference. Abbreviations: SD, Standard deviation; NSAIDs, nonsteroidal anti-inflammatory drugs; IQR, interquartile range; VAS, visual analogue scale; BNI, Barrow Neurological Institute; Gy, gray, N, nerve; T, thalamus. ^a^t-student, ^b^Fisher exact test, ^c^Mann-Whitney U test, ^d^Chi-squared test

Variables	High dose thalamus (n=10)	Low dose thalamus (n=11)	Total (N=21)	P-value
Age in years; mean (SD)	60.4 (17)	61 (18)	60.8 (17)	0.914^a^
Sex; n (%)				
Male	2 (20%)	4 (36.4%)	6 (28.5%)	0.635^b^
Female	8 (80%)	7 (63.6%)	15 (71.4%)	
Duration of pain in months; median (IQR)	96 (90)	84 (51)	84 (87)	0.693^c^
Duration of crisis before treatment in days; mean (SD)	80.5 (35)	69 (17)	65 (28)	0.301^a^
Type of medications used				1.00^d^
Monotherapy; n (%)	5 (50%)	5 (45.5%)	10 (47.6%)	
NSAIDs	0	1 (20%)		
Opioids	3 (60%)	3 (60%)		
Neuromodulators	2 (40%)	1 (20%)		
Combined medications; n (%)	5 (50%)	6 (54.5%)	11 (52.3%)	
NSAIDs plus opioids	3 (60%)	1 (17%)		
NSAIDs plus neuromodulators	0	1 (17%)		
Opioids plus neuromodulators	1 (20%)	1 (17%)		
All	1 (20%)	3 (50%)		
Previous treatments; n (%)				0.635^b^
None	3 (30%)	2 (18.2%)	5 (23.8%)	
Percutaneous or surgical procedures	7 (70%)	9 (81.8%)	16 (76.2%)	
Affected side; n (%)				0.080^b^
Right	8 (80%)	4 (36.4%)		
Left	2 (20%)	7 (63.6%)		
Dose to the thalamus (Gy); median (IQR)	140 (20)	90 (24)		<.001^c^
Dose to the nerve (Gy); median (IQR)	90 (5)	80 (1)		0.002^c^
VAS prior to treatment; median (IQR)	10 (0)	10 (1)	10 (0)	0.823^c^
Vascular conflict detected				1.00^b^
No	3 (30%)	5 (45.5%)	8 (38.1%)	
Yes	7 (70%)	6 (54.5%)	13 (61.9%)	
BNI prior to treatment; n (%)				0.201^d^
IV	3 (30%)	7 (63.6%)		
V	7 (70%)	4 (36.4%)		

**Figure 2:**
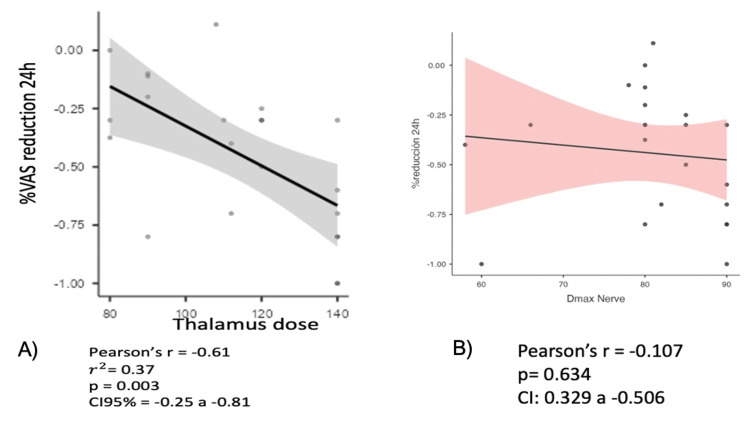
(A) Correlation between thalamus dose (Gy) with percent reduction of VAS score and (B) between nerve dose with percent reduction of VAS score at 24 hours after treatment Abbreviation: VAS, visual analog scale.

The thalamus dose in total population showed a moderate negative correlation with the percentage reduction in VAS score in the first 24 hours (r=-0.61; p=0.003), 48 ​​hours (r=-0.060; p=0.003), 72 hours (r=-0.58; p=0.033), and 96 hours (r=-0.67; p=<0.01) after treatment. Concerning the nerve, the same statistical calculation was carried out for the same period in the first 24 hours (r=-0.107; p=0.634), 48 ​​hours (r=-0.227; p=0.310), 72 hours (r=-0.249; p=0.263), and 96 hours (r=-0.206; p=0.359) after the treatment; however, no significant correlation was observed (Figures 2A, 2B).

**Figure 3:**
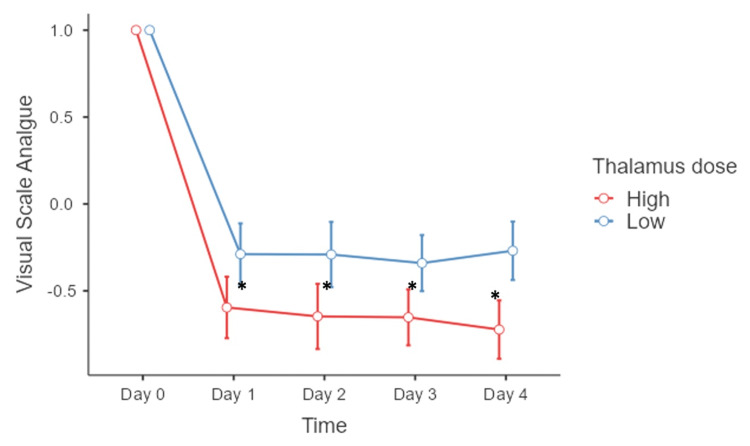
Percentage of reduction of VAS score in the first four days after treatment *Statistical significance between groups (p<0.05) Abbreviation: VAS, visual analog scale.

To compare the effects of the thalamus and trigeminal nerve dose during the first four days after treatment, we conducted a repeated measures ANOVA with Greenhouse-Geisser correction (Mauchly’s test p=0.002; Figure 3). Only the dose to the thalamus (F(1,18) = 7.7, p=0.013) and its interaction with time (F(2.65,9) = 156, p=0.029) was statistically significant, while dose delivered to the nerve did not show a significant correlation (F(1,18) = 0.01, p=0.935). The global average reduction in VAS score for the high-dose group was -65% during the evaluated period, while for the low-dose group the global average reduction was -30%, suggesting that the low-dose group did not exhibit a radioneuromodulation effect according to our definition.

**Figure 4:**
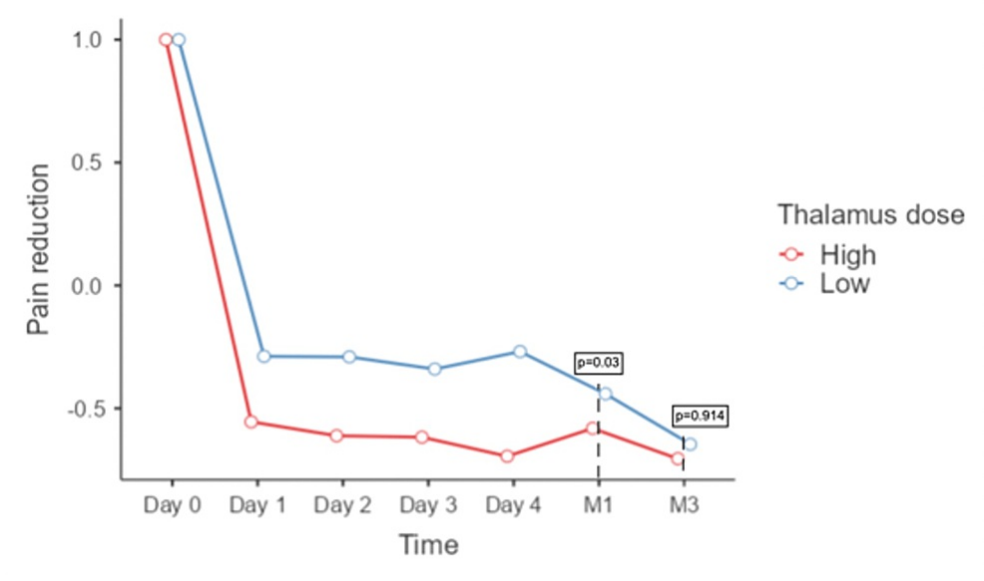
Pain reduction after irradiation to the nerve and thalamus according to the dose delivered to the thalamus in the first five days, at one and three months

Following the post-hoc analysis, we found statistically significant differences between the groups five days after treatment. The high thalamus dose group exhibited progressive radioneuromodulation from the first day (Day 1: -62%; Day 2: -65%; Day 3: -65%; Day 4: -74%). In contrast, the low thalamus dose group did not show radioneuromodulation during the first four days (Day 1: -27%; Day 2: -29%; Day 3: -34%; Day 4: -25%). One month after treatment, the group receiving a higher dose of radiation to the thalamus experienced a greater reduction in pain, as measured by the VAS score, with a mean VAS score of three (range, 2 to 8), compared to the group receiving a lower dose of radiation, with a VAS score of six (range, 0 to 9). This resulted in a -60% reduction in pain for the high-dose group versus a -44% reduction for the low-dose group (p=0.03). At three months, the mean VAS score was 2 (range, 0 to 5) in the high-dose group and 4 (range, 0 to 8) in the low-dose group, but this difference was no longer statistically significant (p=0.91; Figure 4).

We performed the same analysis to evaluate the effect of the two high radiation doses on the thalamus. Repeated ANOVA measures revealed a global reduction of -76% with 140 Gy and -46% with 120 Gy, but this was not statistically significant [F(1,8)=4.7, p=0.060]. However, high thalamus doses and their interaction with time were statistically significant (F(4,32)=4.20, p=0.008). We did not use the Greenhouse-Geisser correction in this case (Mauchly test, p=0.277). In the post-hoc analysis, patients who received 140 Gy in the thalamus, achieved radioneuromodulation from the first day (Day 1: -75%; Day 2: -79%; Day 3: -76%; Day 4: -75%), while patients who received 120 Gy exhibited radioneuromodulation until Day 4 (Day 1: -33%; Day 2: -40%; Day 3: -46%; Day 4: -68%). The difference between groups was significant only for Day 1 and Day 2. At one month, the percentage reduction based on VAS showed that patients who received 140 Gy had a reduction of -68%, while patients with 120 Gy or less had a reduction of -40%, which was a significant difference (independent sample t-test p=0.03; Figure 4).

Biologically effective dose

Comparing the BED of GK vs the BED from CK regarding radioneuromodulation did not yield significant findings. The mean BED for patients treated with GK who exhibited a radioneuromodulatory effect was 3,666.02 Gy2.47 (equivalent to 94 Gy approximately in a single fraction), while the BED for patients who did not show a radioneuromodulatory effect and were treated with CK was 3,292.835 Gy2.47 (equivalent to 89 Gy in a single fraction). These values were not significantly different (t-test, p=0.23) despite the wide variation in physical dose and the different radioneuromodulatory responses. Table 2 summarizes characteristics regarding dose, BED, radioneuromodulation and technology in each patient.

**Table 2:** Characteristics regarding dose, BED, radioneuromodulation and technology in each patient *DMax: Maximum dose. Abbreviations: Gy, gray; BED, Biologically Effective Dose; GK, GammaKnife (Infini); CK, Cyberknife.

Patient	Technology	Nerve dose (Gy)	Thalamus dose (Gy)	BED thalamus (Gy_2.47_)	Equivalent Dose Thalamus	Time of treatment to the thalamus (seconds)	Radiomodulation
1	GK	90	140	4,418.5	103.2	6,816	Yes
2	GK	90	140	4,386.9	102.8	6,970	Yes
3	GK	90	140	4,332.9	102.2	7,242	Yes
4	GK	90	140	4,300	101.8	7,413	Yes
5	GK	90	140	4,358.4	102.5	7,112	Yes
6	GK	90	140	4,352.1	102.4	7,144	Yes
7	GK	80	120	3,305.7	89.1	6,544	Yes
8	GK	85	120	3,175.5	87.3	7,419	Yes
9	GK	85	120	3,191.1	87.5	7,308	Yes
10	GK	85	120	3,183.9	87.4	7,359	Yes
11	GK	80	80	1,649	62.6	4,710	Yes
12	GK	80	80	1,684.4	63.3	4,366	No
13	GK	80	80	1,630.5	62.2	4,900	No
14	CK	80*	90	2,389.4	75.6	2,760	Yes
15	GK	80	90	2,028.6	69.6	5,100	No
16	CK	78*	90	2,511.4	77.5	2,220	No
17	GK	80	90	2,019	69.4	5,184	No
18	CK	80	110	3,752.9	95.1	2,520	Yes
19	CK	81*	110	3,553.1	92.5	2,340	No
20	CK	78*	110	3,598.5	93.1	3,000	No
21	CK	76*	110	3,508.3	91.9	2,880	No

**Figure 5:**
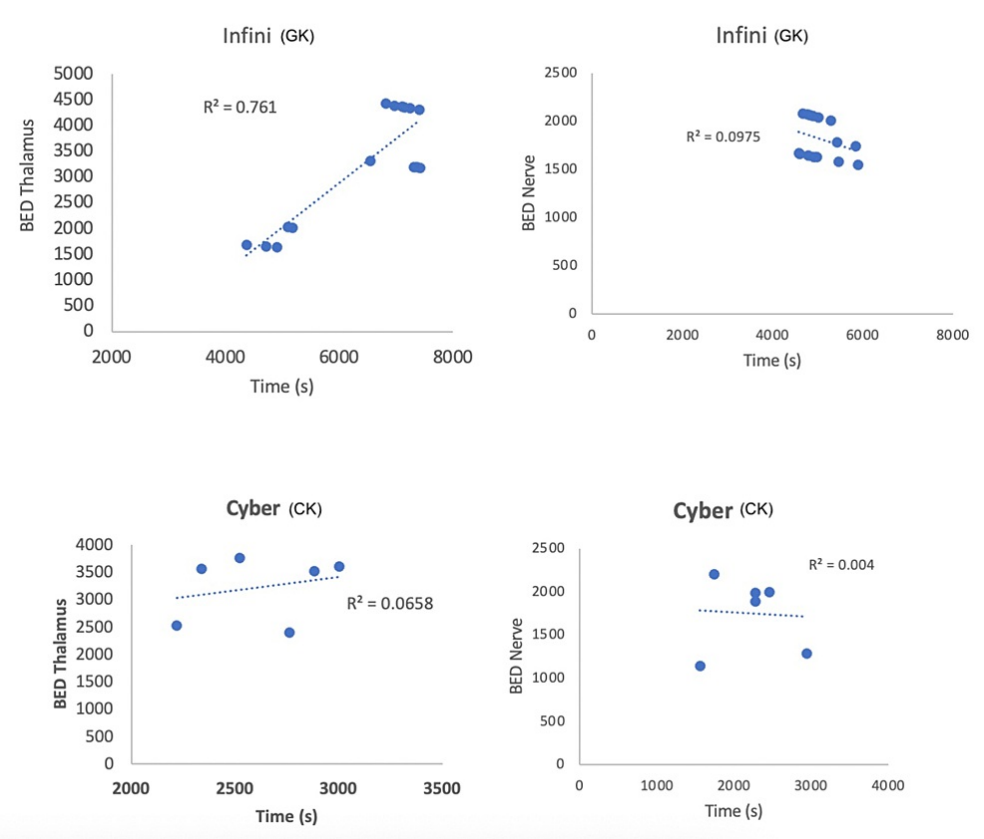
Correlation between biologically effective dose (BED) to the nerve and Cyberknife (CK) versus Infini (GK)

**Figure 6:**
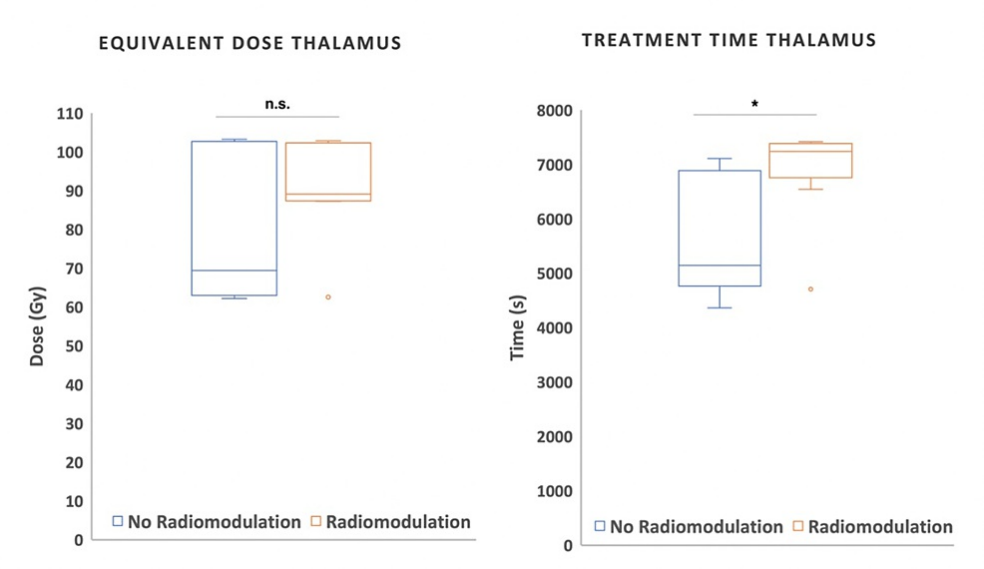
Equivalent dose and treatment time to the thalamus for patients treated with Infini (GK)

A positive correlation exists between treatment time and BED delivered to the thalamus in patients treated with GK, where a longer treatment time correlates with a higher BED, as expected due to a prescribed higher physical dose (Pearson correlation, r=0.87, p=<.001). As a result, the treatment time was higher for patients who exhibited radioneuromodulation (unpaired t-test, p=0.011). This correlation was not observed for the nerve nor for the CK (Figure 5). In terms of BED and equivalent dose, there was no statistically significant difference between patients treated with GK showing radioneuromodulation versus those who did not (unpaired t-test, p=0.08, Table 3). Treatment time was the only variable that significantly differed between patients treated with GK with and without radiomodulation (Figure 6).

**Table 3:** Biologically effective dose (BED) and radioneuromodulation *t-test unpaired samples

Radiomodulation	BED Thalamus	BED Nerve	Equivalent Dose Thalamus	Equivalent Dose Nerve	Treatment time (s) Thalamus	Treatment time (s) Nerve
Yes (n=9)	3,541.9 ± 907.96	1,843.14 ± 216.23	91.49 ± 13.04	66.14 ± 3.97	6,901 ± 867.02	5,125.6 ± 488.05
No (n=6)	2,689.90 ± 1,326.10	1,763.16 ± 212.04	78.37 ± 19.22	64.61 ± 3.94	5,579.6 ± 1113.38	4,979 ± 332.033
P value*	0.080	0.24	0.068	0.23	0.011	0.26

Regarding QoL, a high dose to the thalamus resulted in a better QoL index in the first 4 days after treatment (86%) compared to a low dose to the thalamus (63%), leading to a significant difference between the groups (repeated measures ANOVA, p=0.02). After a month, the QoL index was 90% and 80%, respectively (independent sample t-test, p=0.04). The high-dose nerve-thalamus group exhibited a more pronounced reduction, with a stable -70% reduction in the first four days and a -55% reduction after one month. The low-dose nerve-thalamus group did not have such a significant reduction and reached a reduction of -44% for the month.

**Figure 7:**
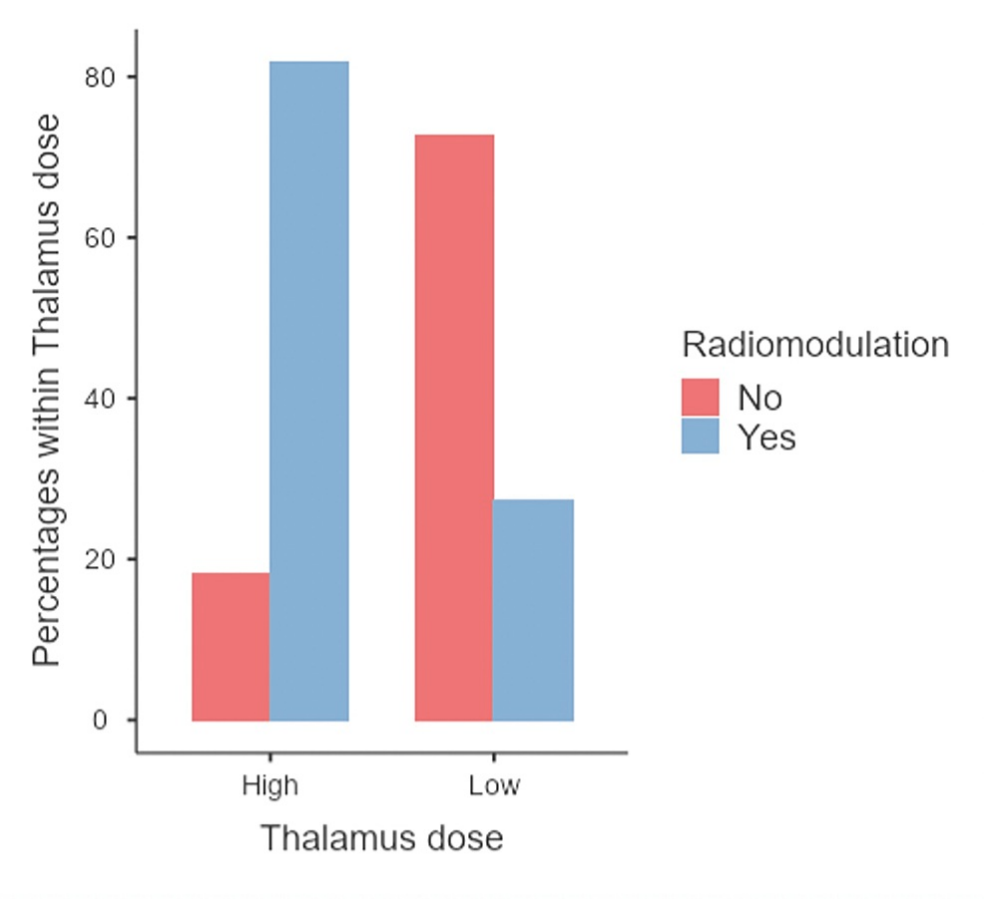
Comparison of radioneuromodulation between high- and low-dose thalamus groups

When comparing high and low doses of the thalamus with radioneuromodulation, 81.8% of patients in the high-dose group exhibited radioneuromodulation, while 27.3% in the low-dose group did (Chi-squared test p=0.010; Figure 7). The high-dose group primarily received treatment with Infini (91%), and one (9%) received treatment with CK; in the low-dose group, six (55%) received treatment with Infini, and five (45%) with CK. Among those who received a high dose to the thalamus, they achieved 100% radioneuromodulation.

As of the final follow-up, the high-dose group had a mean follow-up of 437 days (range, 55 to 1,182 days) and a means VAS score of 2.2 (range, 0 to 7). For the low-dose group, the mean follow-up was 216 days (range, 142 to 344 days), and the mean VAS score was 2.9 (range, 0 to 6).

Adverse effects

**Figure 8:**
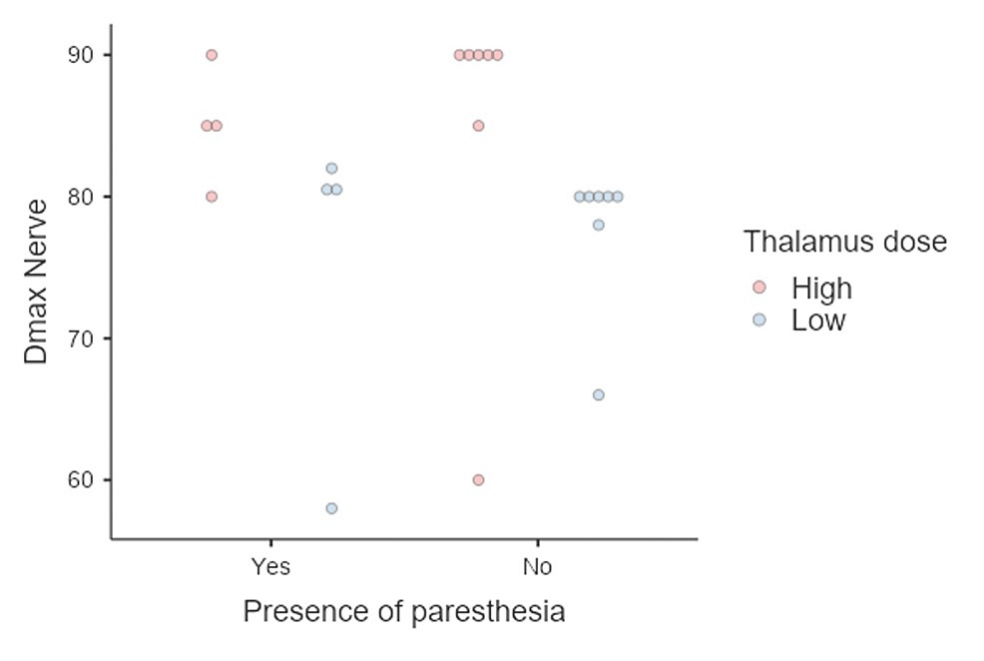
The presence of paresthesia according to the dose given to the nerve

At a median follow-up of 11 months for the whole series the incidence of paresthesia was 36% of the total population, all categorized as mild facial numbness that was not considered bothersome. The anesthesia was unrelated to the dose administered to the trigeminal nerve or thalamus (Figure 8). No additional adverse effects were seen among patients in this study.

## Discussion

Radioneuromodulation with RS is well established, and its beneficial effects, like seizure reduction, pain relief, and tremor improvement, do not always stem from necrosis. Regis et al. observed clinical seizure reductions in arteriovenous malformations (AVM) patients treated with RS before occlusion [17]. Some propose a “cockade” model, featuring irradiated necrosis, a sub-necrotic layer, and an outer radioneuromodulatory region [18].

Larsson et al. found a relationship between higher doses and faster necrosis development [19]. Radiation necrosis occurs within three months at doses above 130 Gy and within three to four weeks at 200 Gy. Chemical changes, such as reduced acetylcholine, may contribute to seizure reduction in AVM patients [17].

Recent animal research demonstrates lower radiation doses and differing radiation tolerance between grey and white matter, with the white matter being more sensitive [20]. Immediate radiation effects range from the first seconds to days, involving changes in enzyme activity and reduced synaptic transmission in animal models [21].

Even though radioneuromodulation is an ill-defined term it is usually associated with a neuronal circuitry response at various doses that might be either ablative or not depending on the indication and volume; nevertheless, some authors associate it with a lower dose spectrum. For example, Regis et al. [17] report this phenomenon as an improvement of seizures while irradiating AVMs and although the doses prescribed are substantially lower than the ones delivered in a typical functional case, the volume receiving doses known to produce necrosis, a volume larger than 12 Gy might be substantial.

We argue that substantial pain relief achieved in hours to a few days after treatment alters the neuronal circuitry as it traduces into clinical benefit much earlier than necrosis occurs using an ablative dose, thus for us a radioneuromodulatory effect occurs in pain regardless that the dose could be considered ablative or not. A classic example of this happens when a high dose of radiation to the hypophysis is delivered in cancer patients, usually, pain relief is measured in hours or days, while clinical evidence demonstrates that the hypophysis continues to function regardless of the ablative dose being delivered.

Evidence suggests that chronic neuropathic pain patients have abnormally high neuronal firing in the CM and centrolateral nucleus of the thalamus [22]. Slowing neuronal activity in these areas after high radiation exposure may partly explain the radioneuromodulatory effects resulting in rapid pain relief.

Understanding the appropriate dose and targeting for clinically effective pain reduction is limited. The CM nucleus, covered by the 50% isodose line, serves as a relay for the paleospinothalamic tract and may mediate the affective component of pain [22]. It is important for TN or complex facial pain, as the CM and PFc receive afferents from various tracts and the trigeminal lemniscus [23,24].

A high radiation dose (90-140 Gy) delivered with a small collimator (4 mm) using GK to targets like the CM and PFc produces larger areas of influence. The 20 Gy isodose line causes a significant decrease in epileptiform spiking in animal models [25]. The areas of influence vary between technologies, such as GK and CK, providing a wider area of influence in different thalamic nuclei that possibly yield different clinical results [9,26,27].

Appropriate dose and targeting, collimator size correlation, optimal volume, and the BED impact remain unclear. BED considers factors like dose rate and treatment time, which can be affected by involuntary breaks during patient treatment. In this series, BED alone was not found relevant for radioneuromodulation due to the small sample size, nevertheless comparing CK and GK, clinical results favored GK despite similar BEDs, suggesting BED alone might not be the only factor. Analysing GK alone, only longer treatment time was significant.

The relationship between decreasing dose rate due to cobalt decay, longer treatment time at the same physical dose, and lower BED needs further understanding. Patients exhibiting radioneuromodulation effects with higher physical doses like 140Gy, display higher BEDs as they are co-dependent despite longer delivery times. However, the limitations of this small retrospective series, including the disproportion of patients treated with GK compared to CK, make drawing solid conclusions difficult.

An important question arises: Do extended treatment time and exposure to a relatively low dose rate favor radioneuromodulation? More studies on BED in functional RS, comparing dose rates and treatment times, and technologies, are required to fully comprehend its clinical significance. Standardizing treatment recommendations based on BED and adjusting the physical dose to the technology used or cobalt decay is crucial if possible. 

TN pain crises and CCP have traditionally been beyond the scope of RS, requiring urgent care and intervention. A single target approach to the nerve typically needs significant time for pain relief. In our TN case series comparing distal or proximal targeting of the dorsal root entry zone (DREZ), eight patients (16.3%) experienced a radioneuromodulation effect on pain, mostly with a more distal target of DREZ [28]. Building on this experience and thalamus radiation in refractory TN [29], a dual strategy approach was created for difficult CCP and refractory pain crisis cases, aiming for rapid pain relief using 140 Gy to the thalamus and 90 Gy to the nerve [8]. This series prompted dose reduction to achieve the lowest effective dose, ideally within the realm of a sub-necrotic dose.

Our results support a higher thalamic dose in the dual target strategy for treating CCP TN pain crises, leading to improved QoL due to rapid pain relief, specially at 140 Gy. High-dose patients showed better pain control at one month than low-dose patients, with a non-statistically significant difference after three months that slightly favored high doses, warranting a larger study. In both oncological and non-oncological pain cases, we observed pain crises after successful radioneuromodulation. These spikes vary in intensity and duration for TN but tend to stabilize over time. Oral phenytoin and prednisone helped alleviate pain during crises lasting 72 hours to seven days.

Safety and complications showed that non-bothersome paresthesia was comparable to single-target RS to the nerve, with 36% of dual-target cases and 35.3% of single-target distal cases [28]. Paresthesia in the dual-target approach likely results from nerve irradiation rather than thalamic irradiation, as the thalamic target seems complication-free in contemporary series like Urgosik’s [30] and our own.

## Conclusions

Our findings indicate that high-dose dual-target RS yields more consistent radiomodulation in CCP and TN than single-target irradiation. However, low-dose thalamic dual-target strategies for TN did not provide consistent pain relief, leading our group to discontinue this approach. Understanding BED's significance in functional RS and potential physical dose adjustments based on technology or cobalt decay is essential for standardizing treatment recommendations. Though the relationship with BED, remains unproven by our series, longer treatment times and higher physical doses appear relevant, warranting further clinical evidence for different technologies and dose rates.

Higher thalamic doses in a dual-target strategy may improve outcomes for TN pain crises and CCP patients. Further studies should validate this approach for treating refractory CCP in TN and other complex facial pain, investigate treatment safety and potential complications, and assess long-term effects on patient outcomes and QoL.
